# Bibliographical Department

**Published:** 1880-03

**Authors:** 


### BIBLIOGRAPHICAL DEPARTMENT.

We are indebted to Dr. Laurence Turnbull, of Philadelphia, for a copy of his book entitled "The Advantages and Accidents of Artificial Anæsthesia." A manual of anæsthetic agents and their employment in the treatment of disease. By Laurence Turnbull, M. D., Ph. G., Aural Surgeon to Jefferson Medical College Hospital; Physician to the Department of Disease of the Eye and Ear, Howard Hospital, Philadelphia; Fellow of the American Association for the Advancement of Science; and of the American Medical Association, etc. Honorary Member of the Maryland and District of Columbia Dental Association; and of the Meigs and Mason Medical Society of Ohio. The book is well written, and contains about all that is important to be known upon the subject upon which it treats. Our copy is the second edition of the work. It is well illustrated, and is published in very nice style by Messrs. Lindsay & Blackistone, of Philadelphia.

Through the courtesy of our energetic and efficient Health Commissioner, Dr. James A. Steuart, we have been placed in possession of his very excellent "Annual Report of the Health Department, to the Mayor and City Council of Baltimore, for the twelve months ending December 31, 1879." The report is full and explicit on the subjects of which it treats, and shows conclusively that Dr. Steuart is "the right man in the right place," as the head of the Health Department of Baltimore.

Dr. T. W. Jones, of Columbus, O., has kindly sent us a book of the "Transactions of the 34th Annual Meeting of the Ohio State Medical Society," held at Dayton, June 3, 4, and 5, 1879. The report embraces a number of able papers from some of the most eminent medical men of the West, and we would gladly copy from its pages on several subjects did our space allow of our doing so. We cannot refrain from saying, however, that Dr. I. F. Baldwin's able paper on "The Metric System" meets our hearty endorsement. In regard to the proposed change, his statements and arguments are unanswerable. Our decided opinion is to "let well enough alone."

"The Physician and Bulletin of the Medico-Legal Society." A monthly journal, devoted to practical medicine and medical jurisprudence. Editors of the medical department, E. H. M. Sell, A. M., M. D., and H. P. Gislance, M. B. Editor of the department of medical jurisprudence, Geo. W. Wells, A. M., M. D. New York. Subscription, $2.00 per annum. Is a well-edited and excellent monthly.

"The College and Clinical Record." A monthly medical journal, conducted especially in the interests of the graduates and students of Jefferson Medical College. Edited by Richard J. Dunglison, M. D., and Frank Woodberry, M. D. Subscription, $2.00, in advance. It will doubtless be found to fulfill the objects for which it was established, under the editorial management of its accomplished editors. We wish it success.

"The Medical Herald," is another medical journal that has reached us from Louisville, Ky. It possesses some very good reading matter, and appears in a plain but neat dress. It is edited by Drs. Dudley S. Reynolds and I. M. Mathews, both of the Hospital College of Medicine.

"The American Medical Journal" has been received. It seems to be published in the interest of the Eclectic School of Medicine, but it contains a number of very good orthodox articles. Edited by Dr. George C. Pitzer, and published in St. Louis, at $2.00 per annum.

"The Southern Clinic." A monthly journal of medicine, surgery, and new remedies. C. A. Bryce, M. D., editor and proprietor. Is published in Richmond, Va. $1.50 per annum, in advance. This is a sprightly and interesting journal of 31 pages reading matter.

"The Kansas Medical Index," is a new medical journal, devoted to medicine, surgery and allied sciences. F. F. Dirkman, M. D., editor. 28 pages; monthly; $1.50 per annum. We wish it success.

Number 9 of the "Country Practitioner, or New Jersey Journal of Medical and Surgical Practice." Published monthly at Beverly, Burlington county, N. J. E. P. Townsend, M. D., editor. $2.00 per annum; 33 pages. Quite interesting in its original and judiciously selected matter. New Jersey ought to support one journal very handsomely.

"The Proceedings of the Medical Society of Kings County." Conducted by the Council, and published in Brooklyn, N. Y. Is regularly received, and is always a welcome guest. Price, $2.00 per annum.

We have been greeted for the past three weeks by our whilom friend, the "Medical and Surgical Reporter," of Philadelphia, and its pages seem to have lost nothing in value and variety since the days of its founder and able editor, Dr. Samuel W. Butler. We hope it may continue to increase in value as it grows in years. Published weekly at $5.00 per annum. D. G. Brinton, M. D., editor.

"The Medical Brief." A monthly journal of practical medicine. Is published in St. Louis, Mo., at $1.00 a year, in advance, by Dr. I. I. Laurence, editor and proprietor. It is *multum in parvo*, and will be found useful to many practitioners.

We are pleased to receive No. I. Vol. I, of "Die Zahntechnische Reform," edited and published by Gustav H. Pawley, Berlin, Germany.

"The Obstetric Gazette." A monthly journal, devoted to obstetrics, with diseases of women and children. Edward B. Stephens, A. M., M. D., editor. Terms, $3.00 yearly, in advance. Is an excellently edited and valuable journal on the subjects to which it is devoted.

"The Clinical News." A national weekly journal of clinical medicine, surgery and gynecology. Philadelphia. Price, $3.00 a year, postpaid. Samuel M. Miller, M. D., editor and publisher. Contains much valuable information, gleaned weekly from the lectures in the hospitals and charitable institutions of Philadelphia.

In addition to the foregoing, the following have been received as this copy is going to the printer: "The Medico-Literary Journal." By Mrs. M. P. Sawtelle, M. D., San Francisco. A monthly journal; $3.00 a year, in advance. And "The New Medical Eclectic." Monthly; $2.00 per annum. Editors, Drs. Robert S. Newton, Sr. and Jr.

